# Mapping stakeholders and policies in response to deliberate biological events

**DOI:** 10.1016/j.heliyon.2018.e01091

**Published:** 2018-12-27

**Authors:** Rebecca Katz, Ellie Graeden, Keishi Abe, Aurelia Attal-Juncqua, Matthew R. Boyce, Stephanie Eaneff

**Affiliations:** a Georgetown University Center for Global Health Science and Security, 3900 Reservoir Road, NW Washington, DC 20007, USA; b Talus Analytics, Boulder, CO, USA; c Talus Analytics, Boston, MA, USA

**Keywords:** Information science, Public health, Epidemiology, Political science

## Abstract

**Background:**

Recent infectious disease outbreaks have brought increased attention to the need to strengthen global capacity to prevent, detect, and respond to natural biological threats. However, deliberate biological events also represent a significant global threat, but have received relatively little attention. While the Biological Weapons Convention provides a foundation for the response to deliberate biological events, the political mechanisms to respond to and recover from such an event are poorly defined.

**Methods:**

We performed an analysis of the epidemiological timeline, the international policies triggered as a notional deliberate biological event unfolds, and the corresponding stakeholders and mandates assigned by each policy.

**Findings:**

The results of this analysis identify a significant gap in both policy and stakeholder mandates: there is no single policy nor stakeholder mandate for leading and coordinating response activities associated with a deliberate biological event. These results were visualized using an open source web-based tool published at https://dbe.talusanalytics.com.

**Interpretation:**

While there are organizations and stakeholders responsible for leading security or public health response, these roles are non-overlapping and are led by organizations not with limited interaction outside such events. The lack of mandates highlights a gap in the mechanisms available to coordinate response and a gap in guidance for managing the response. The results of the analysis corroborate anecdotal evidence from stakeholder meetings and highlight a critical need and gap in deliberate biological response policy.

## 1 Introduction

The threat of a deliberate biological event is stronger now than ever. Scientific advances, including genomic editing and other dual use research of concern have made the creation of biological weapons more feasible for a wider range of actors. Geopolitical shifts and conflict, including heightened tensions on the Korean peninsula, have also increased the threat of biological weapons use [1]. The use of chemical weapons in Syria, and as assassination tools, have challenged international norms and lowered barriers to use. The risk is compounded by the ever-expanding natural threat, as microbes evolve and emerging infectious diseases threaten population health.

While the world is far from ready, funds and political attention have been marshalled to build national and international capacity to prevent, detect and respond to natural biological threats. Few resources, however, have been devoted to deliberate biological events (DBE). The majority of the response capacities required for a naturally occurring biological event will also apply to a DBE, including the need to detect cases, treat patients, identify the pathogen, and contain the spread of disease [2]. However, many of the standard practices and the stakeholders involved in outbreak response activities may be hindered or altered given the malicious nature of a deliberate event. DBEs introduce heightened requirements for responder safety and security, with implications ranging from life insurance requirements, to the need for additional security personnel, to potential restrictions at points of entry. Concurrent criminal investigations, fact-finding efforts, and evidence collection can complicate humanitarian response efforts, potentially restricting access for emergency response personnel, preventing data sharing, and altering the chain of command. Indeed, many traditional stakeholders may not have plans or mandates to engage in the response to a DBE, leaving the international community at significant risk of a slow and ineffective response and recovery strategy.

The Biological and Toxins Weapons Convention (BWC), under Article VII, commits “each [State Party] to provide or support assistance, in accordance with the United Nations Charter, to any Party to the Convention which so requests, if the Security Council decides that such Party has been exposed to danger as a result of violation of the Convention.” [3] Yet, operationalizing this article has several significant challenges. First, unlike the Chemical Weapons Convention, there is no Secretariat capable of investigating and coordinating a response to a DBE. Second, although the BWC has been in force for over 40 years, there is still no documented procedure or plan for requesting or providing assistance in response to a violation of the treaty. Third, the actors that would be involved in the response to a DBE and their associated mandates are poorly understood by States Parties to the BWC, to such an extent that it remains unclear how a response might be coordinated, or which parties would be available to come to the assistance of an affected population. Indeed, even determining whether or not a biological event is naturally occurring or deliberate can be a monumental task [2], adding to the complexity of rapidly understanding and implementing the policies that govern DBE response [4].

In September 2017, a gathering of international experts at Wilton Park agreed upon the need to define and map stakeholders and clarify processes associated with BWC Article VII to enhance the international capacity to respond to DBEs [5]. In response, our research team identified stakeholders, relevant policies, and roles in a deliberate event scenario and mapped each on a timeline of a notional biological outbreak scenario affecting humans and suspected to be deliberate. This effort was informed by ongoing work by both the United Nations Office of Counter-Terrorism (OCT), as well as the Implementation Support Unit of the Biological and Toxin Weapons Convention Article VII project [6]. Based on these data, we created a series of interactive tools to support decision makers and help elucidate the complex space of identifying and responding to DBEs.

## 2 Methods

### 2.1 Identification of stakeholders, roles, and relevant policies

Following discussions with an international panel of experts at Wilton Park in September 2017, we conducted structured literature reviews to identify stakeholders with roles in responding to a biological outbreak suspected to be deliberate, and the policies that govern each stakeholder's role. Peer-reviewed and grey literature searches included searches of PubMed, JSTOR, Google Scholar, UN agency websites, and web presences of other international organizations. Multiple researchers collaborated on screening materials and discrepant opinions were discussed amongst all authors until a consensus was reached.

### 2.2 Development of data ontology and DBE stakeholder database

To capture and reflect the data gathered through workshops, interviews, and the literature review, the research team developed a data ontology to link stakeholders to the policies and mandates that define and support their functional roles through a DBE. These data were aligned with decision points on a notional event timeline to identify the policies relevant during each sub-event on the timeline. An ontology was defined to link each policy to its corresponding trigger during a notional event timeline and to the stakeholders mandated to respond based on each policy.

Stakeholders are those organizations, agencies, or groups expected to be involved in identifying, responding to, or recovering from a DBE, including affected and non-affected national governments, United Nations (UN) organizations, other international organizations (IOs), local and international non-governmental organizations (NGOs), and private sector organizations. These stakeholders are governed by policies that define mandates over the course of an unfolding DBE, and include constitutions, resolutions, conventions, memorandums of understanding, and mission statements. These mandates define stakeholder roles across four sectors: public health and medical, humanitarian assistance, safety and security, or governance and policy.

We custom built a stakeholder database around the DBE ontology with each policy mandate extracted and mapped to the stakeholder(s) and to the event on a notional timeline to which it is relevant. Based on the policy mandates, stakeholders were categorized by their primary and secondary (if any) roles in the response timeline. In total, we identified 56 policies and 52 stakeholders in DBE response and incorporated them into the DBE stakeholder database. Additional information, including references, hyperlinks for each policy and a brief written summary description of each stakeholder's role during a DBE, are also included in the database and can be accessed online via the DBE stakeholder tool.

### 2.3 Online DBE stakeholder visualization

We organized the stakeholder and mandate data into a DBE stakeholder tool, available online (https://dbe.talusanalytics.com). The online tool describes how a deliberate biological outbreak might unfold; defines the stakeholders who would be involved in identifying, responding to, and/or recovering from the event; and aligns these events and stakeholders with the policies governing these efforts. This information is visualized through a web-based user interface built in HTML5 and JavaScript. The data are drawn from a custom-built database populated by the research team, as described above.

The online tool allows users to navigate through a notional event timeline to identify stakeholders, roles, and corresponding policy mandates for each timeline event. At each point in the event timeline, policies are identified alongside the stakeholders who draw their mandates from each policy. Policies are identified as they become applicable across the timeline. Hyperlinks can be used to access all publicly-available policies, curated in an online document library.

## 3 Results

**Fig. 1:**
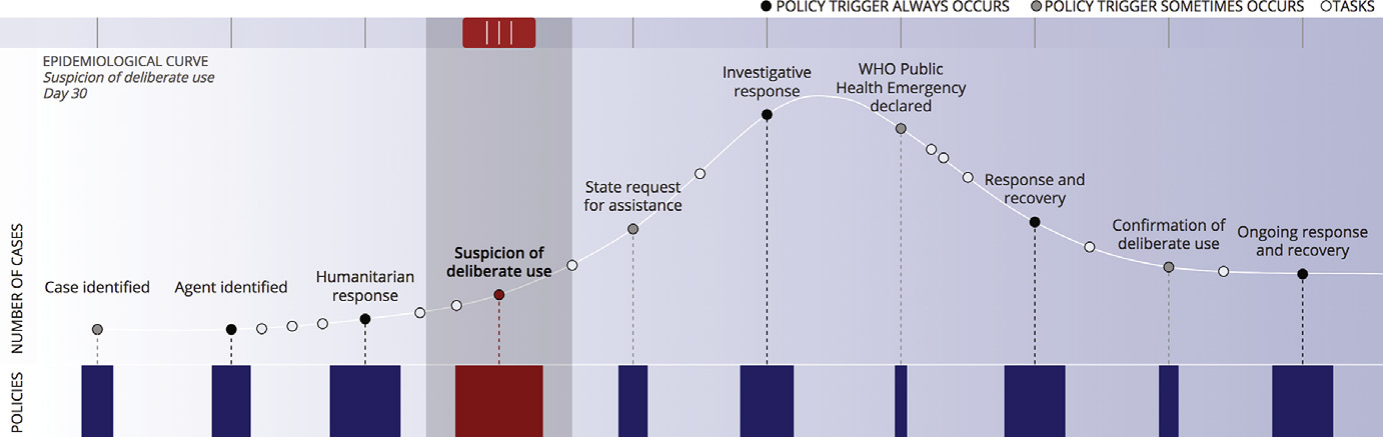
Timeline of a deliberate biological event describing triggers for policy mandates and stakeholder engagement.

Deliberate biological events are driven by many of the same factors as natural biological outbreaks. As with any outbreak, the initial events relate to the outbreak itself: identify cases, characterizing the causative agent, and initiating an epidemiological and public health response. Policies mandating response activities and stakeholder roles are triggered by these events (Table 1). Though the epidemiological and public health response activities associated with naturally-occurring outbreaks are well-practiced [7, 8, 9] and therefore fairly well-defined [10, 11], the policy and stakeholder landscape is significantly less well-characterized for DBEs. As shown in Fig. 1, we have analyzed the policies triggered by each event along a DBE timeline and characterized the stakeholders and their mandated roles in those events. While the specific order and location of the sub-events along the timeline will differ between DBEs, the overarching pattern of the notional event provides context for the pattern in policy triggers and stakeholder engagement.

**Table 1:** Policy documents and other mandates detailing the roles and responsibilities of NGOs, IOs and UN agencies in responding to a DBE.

Document name	Year published
*Non-governmental Organizations*	
International Conferences of the Red Cross Commentary on the First Geneva Convention	1949
Médecins Sans Frontières Charter	1971
Resolutions of the International Conferences of the Red Cross and Red Crescent Societies	2011
*Non-United Nations International Organizations*	
Chemical Weapons Convention, Articles IX-X	1997
INTERPOL Constitution	1956
Inter-Agency Standing Committee Framework on Durable Solutions for Internally Displaced Persons	2010
International Organization for Migration Constitution	1954
World Organization for Animal Health Organic Statutes	1924
World Bank Pandemic Emergency Financing Facility Framework	2017
*United Nations*	
Biological Weapons Convention	1975
Biological Weapons Convention Conference VI	2006
Convention to Establish a Customs Co-operation Council	1952
Constitution of the Food & Agriculture Organization of the United Nations	1945
Cooperative Arrangement for the Prevention of Spread of Communicable Disease through Air Travel	2007
Inter-Agency Standing Committee Framework on Infectious Diseases	2016
International Health Regulations	2005
Memorandum of Understanding, World Health Organization & the United Nations	2011
Memorandum of Understanding, World Organization for Animal Health & the United Nations	2012
Milan Plan of Action	1985
United Nations Charter, Chapter VII	1945
United Nations Economic and Social Council Resolution 1989/56	1989
United Nations Institute for Disarmament Research Statute	1985
United Nations International Children's Emergency Fund (UNICEF) Core Commitments for Children in Humanitarian Action	1998
United Nations General Assembly Resolution 204 (III)	1948
United Nations General Assembly Resolution 428 (V)	1949
United Nations General Assembly Resolution 802 (VIII)	1953
United Nations General Assembly Resolution 1714 (XVI)	1961
United Nations General Assembly Resolution 40/32	1985
United Nations General Assembly Resolution 42/37C	1987
United Nations General Assembly Resolution 44/72	1989
United Nations General Assembly Resolution 46/182	1991
United Nations General Assembly Resolution 55/283	2001
United Nations General Assembly Resolution 56/195	2002
United Nations General Assembly Resolution 57/150	2003
United Nations General Assembly Resolution 58/153	2004
United Nations General Assembly Resolution 59/276	2005
United Nations General Assembly Resolution 60/124	2006
United Nations General Assembly Resolution 61/198	2007
United Nations General Assembly Resolutions Establishing UNICEF	1946
United Nations Security Council Resolution 620	1988
United Nations Security Council Resolution 1540^∖^	2004
United Nations Security Council Resolution 1673	2006
United Nations Security Council Resolution 1810	2008
United Nations Security Council Resolution 1977	2011
World Health Assembly Resolution 34.26	1981
World Health Assembly Resolution 46.6	1933
World Health Assembly Resolution 48.2	1995
World Health Assembly Resolution 55.16	2002
World Health Assembly Resolution 58.1	2005
World Health Assembly Resolution 59.22	2006
World Health Assembly Resolution 64.10	2011
World Health Assembly Resolution 65.20	2015
World Health Organization Constitution	1948
World Health Organization Emergency Response Framework	2017

**Fig. 2:**
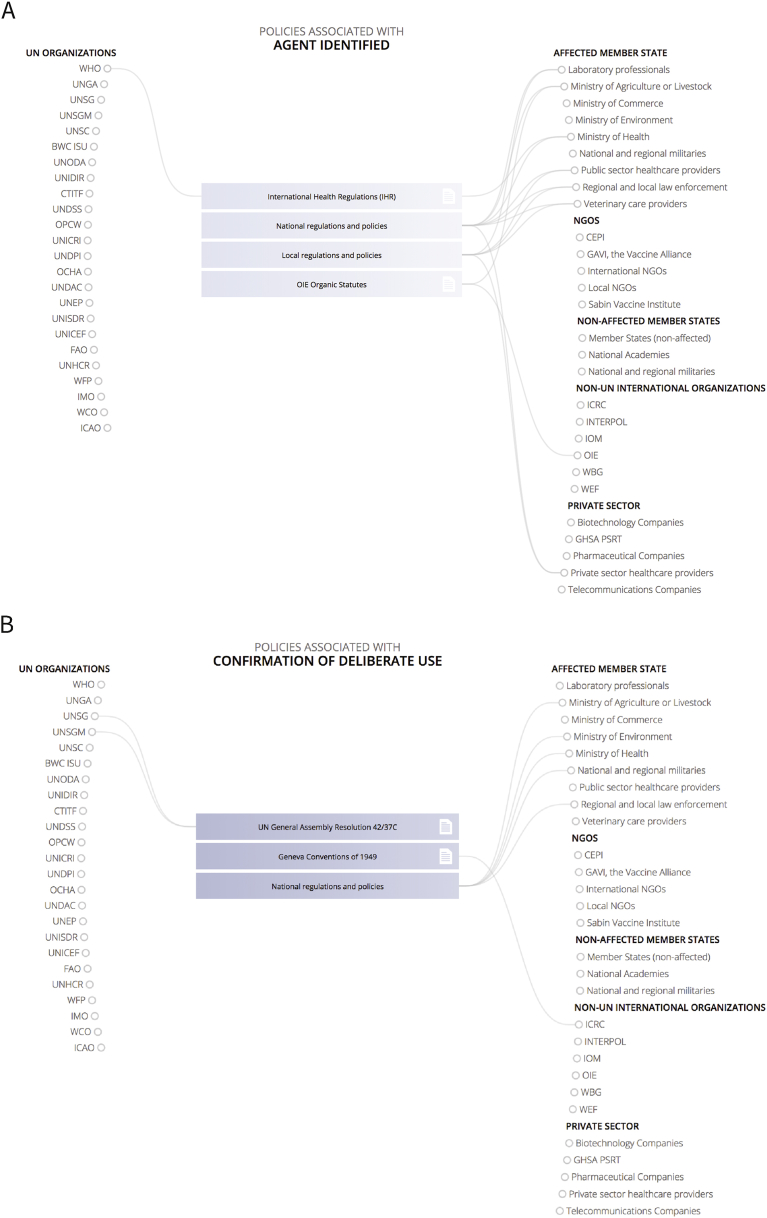
Relevant policies and the stakeholders to whom those policies apply (A) when an agent is first identified, and (B) when the event has been confirmed to be deliberate.

The policies that govern stakeholder roles during event identification, response, and recovery shift as a DBE unfolds. Early response efforts are largely governed by local and national regulations and policies that vary depending on the State in which the outbreak is first identified (Fig. 2A). As the event progresses, additional policies are invoked as UN organizations, other international organizations, non-affected states, NGOs, and private sector organizations become increasingly involved in ongoing event response and recovery. While the greatest number of policies are triggered during the first suspicion of deliberate use, a significant number of policies and corresponding stakeholder mandates and roles are triggered by sub-events that will occur both in naturally-occurring and deliberately-caused outbreaks. When an event is confirmed to have been deliberate, based on the results of epidemiological and criminal investigations, the stakeholders mandated to engage shift from more response or public health-focused organizations to include more governance-based organizations (Fig. 2B).

**Fig. 3:**
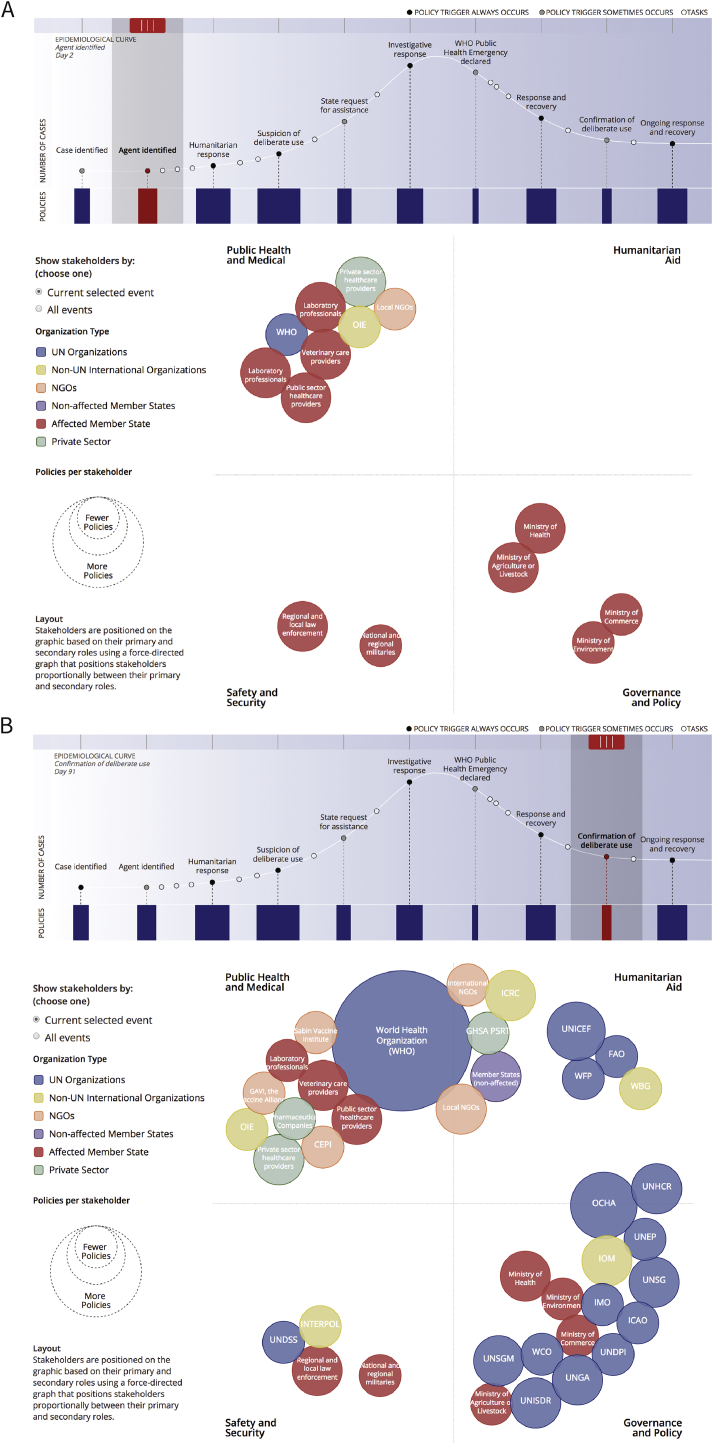
Stakeholders and their functional roles (A) when an agent is first identified and (B) when the event has been confirmed to be deliberate. Stakeholders are sized according to the number of policy documents that identify mandates throughout a deliberate biological event and colored by the type of organization. Each stakeholder is positioned based on their role in the event. Those stakeholders with multiple roles are positioned on the axes centrally between their roles.

As shown in Fig. 3, policies mandate a broad range of organizations to participate. These organizations can be organized based their role(s) across sectors: public health and medical, humanitarian assistance, safety and security, and governance and policy. Early in event response, national ministries of the impacted state, including the ministry of health, and, in the event of a zoonotic outbreak, the ministry of agriculture or livestock, would play a central coordinating role in initial response efforts (Fig. 3A). As an event expands, other international stakeholders play progressively more central roles in response and recovery alongside the impacted state. The World Health Organization (WHO) and the UN Office for the Coordination of Humanitarian Affairs (OCHA) are mandated by a particularly large number of policies and central roles (Fig. 3B).

The number of stakeholders and the number of policies that govern stakeholder involvement grow as the event progresses. Many additional UN organizations and non-UN organizations would begin to play central roles later in the event timeline, particularly in governance and policy and in the provision of humanitarian assistance. While there are many organizations mandated to perform roles in public health and medical or humanitarian assistance, many fewer have roles in safety and security or governance and policy, such as law enforcement and other security organizations. Notably, those with roles in safety and security are more likely to have singular roles and little overlap with those performing roles in public health and medical or humanitarian assistance. This finding confirms those from anecdotal discussions during workshops on DBEs during which many described a lack of formalized mechanisms or policies aimed at supporting coordination between the public health and law enforcement or security organizations.

**Fig. 4:**
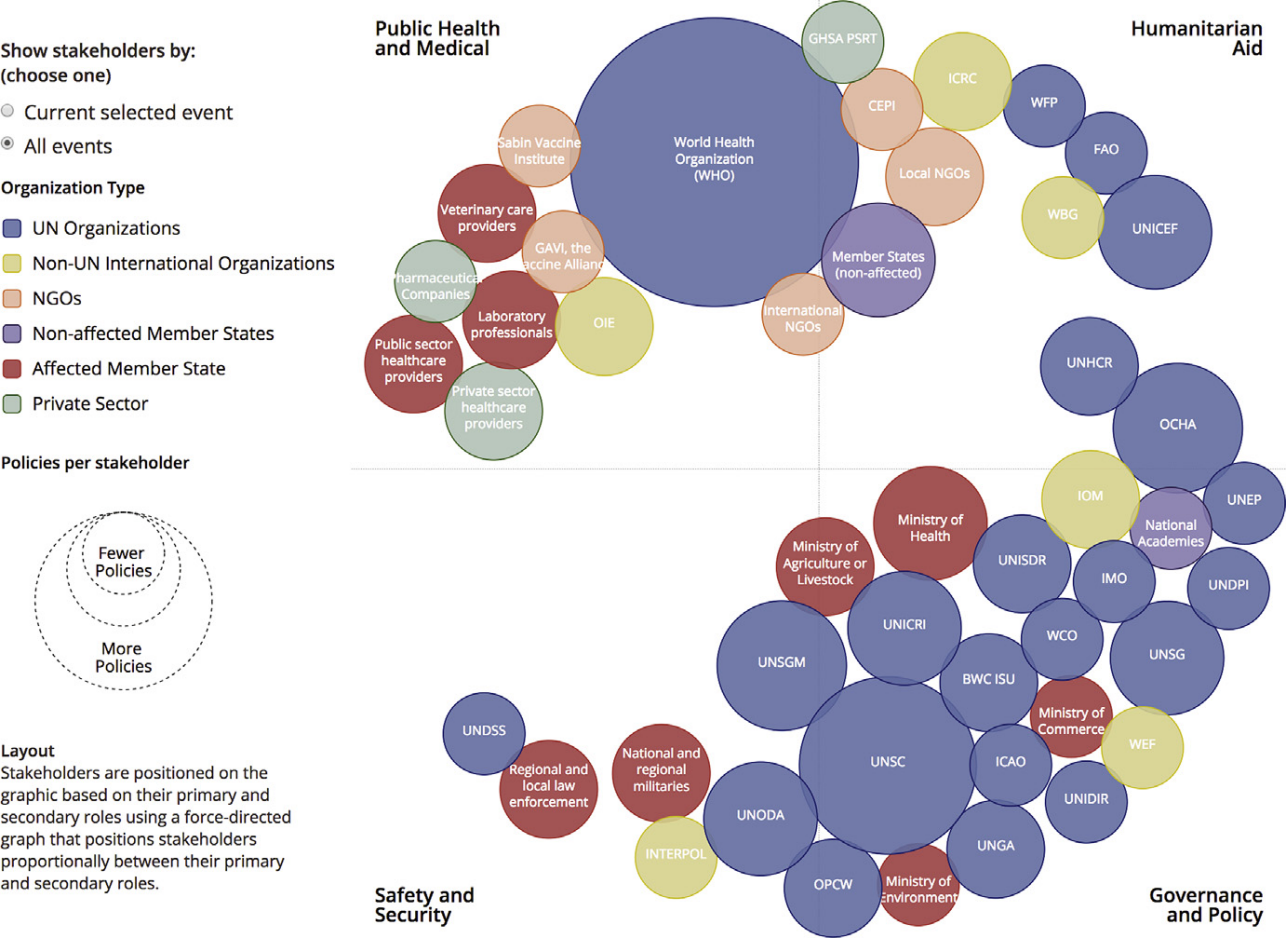
All DBE stakeholders and their functional roles. Stakeholders are sized according to the number of policy documents that identify mandates throughout a deliberate biological event and colored by the type of organization. Each stakeholder is positioned based on their role in the event. Those stakeholders with multiple roles are positioned on the axes centrally between their roles.

Shifting policies and stakeholders across the course of a DBE add to the complexity of response and recovery efforts. While the WHO is broadly mandated to serve a central role, they are mandated to have only a very limited role in safety and security. Indeed, there is no clear single stakeholder with explicit mandates that dictate a central coordinating role in all aspects of event identification, response, and recovery during a DBE. Despite a total list of over fifty identified stakeholders governed by over fifty unique mandates and policy, there is no clear “master” policy mandate governing a coordinated response (Fig. 4).

## 4 Discussion

This paper summarizes and provides a web-based resource for analyzing the intersection between policy, stakeholders, and the epidemiological timeline of a DBE. To the best of our knowledge, this is the first time such an assessment has been completed. This represents a significant step forward in strengthening national and international policies relating to bioterrorism and biowarfare.

Despite the robustness of the global public health response community and the policy framework for naturally-occurring outbreaks, the primary finding from this analysis, as seen in the interactive tool, is that there is no single organization or overarching policy to govern a coordinated, international response to a DBE. Our research addresses the links between stakeholders and the policies that mandate their role; still to be performed is the related analysis identifying and characterizing the second order interactions and linkages between stakeholders that are driven by intersecting, overlapping, or conflicting policies. However, without a single overarching policy providing an integrated governance structure, there are few mechanisms available to guide coordination or to deconflict efforts. Addressing this gap is a critical first step to ensure a successful response to a DBE.

The results presented here define a gap, but also suggest a potential solution. In analyzing the linkages between specific policy mandates and the related stakeholders, the majority of mandates speak to very few stakeholders and almost always only one class of stakeholders (e.g., UN organizations, affected member state, or non-UN international organizations.) To be successful, integrated international policy guidance would require speaking to the vast majority of the stakeholders – both in their roles individually and in their interactions and coordination with other stakeholders. Similarly, the organization tasked with leading and coordinating the response would need to be centrally positioned between all four functional categories of response, with coordination roles in and between public health and medical, humanitarian assistance, governance and policy, and safety and security. The development of such policy guidance would require a collaborative effort amongst national and international stakeholders to clarify and deconflict existing policies, to develop and document clear plans for collaboration and coordination during an event, and to ensure that any policy guidance developed aligns to specific response requirements necessary during a DBE.

A stakeholders' ability to respond effectively to a DBE will be significantly affected by the financial resources available. As a next step in this analysis, we will map how each of the stakeholders are funded – both in peacetime and in emergencies – and how the flow of funds might impact the timing and effectiveness of each stakeholder. Another tool (https://tracking.ghs.org) visualizes funds available to support global health security and could serve as the basis for analysis of the funds globally available for DBE response efforts. This analysis, however, will need to include additional parsing of the data to include specific organizations, beyond the country–level funding currently visualized.

The timeline around which the DBE stakeholder analysis is oriented focuses on the epidemiological and outbreak response timeline. While a critical anchor for considering stakeholder engagement and response activities, there is a parallel timeline of potential military, political, or economic actions in response to suspicion or confirmation of deliberate use. This response could hold significant impacts on the effectiveness of the international response. In addition to addressing the financial constraints of the response, future analyses should focus on defining the military actions and triggers that may impact the ability of some stakeholders to engage or cause conflicting priorities. Understanding how these actions might impact stakeholders, as well as identifying additional stakeholders who may become involved in military, political, or economic actions, is essential for considering the operational response requirements.

## 5 Conclusion

The policy landscape across the DBE timeline highlights that there is no single or unified policy that applies throughout a deliberate biological event, nor any single stakeholder with a clear coordinating role throughout the entire event. While many policies are available to clarify stakeholder roles at a specific point in time, or under specific circumstances, few documents identify an organization's mandates across the entirety of the event timeline. This gap and the lack of a coordinated policy mandate across stakeholders is particularly acute and likely to be problematic during event identification, investigative response, and the associated criminal investigation. Existing policies could potentially be leveraged to provide the badly-needed connective tissue, whether through an updated Inter-Agency Standing Committee (IASC) guidance document giving authorities to a specific UN entity, such as the United Nations Office of Disarmament Affairs, or a revised Health Cluster Guide that specifically addresses DBEs. We hope this tool can be used by decision makers to better understand their roles and authorities during a DBE, identify gaps in capacity and mandates, and move planning efforts forward to create a better prepared world.

## Declarations

### Author contribution statement

Ellie Graeden, Rebecca Katz: Conceived and designed the experiments; Contributed reagents, materials, analysis tools or data; Wrote the paper.

Keishi Abe, Aurelia Attal-Juncqua, Matthew Boyce, Steph Eaneff: Analyzed and interpreted the data; Contributed reagents, materials, analysis tools or data; Wrote the paper.

### Funding statement

This work was supported by a grant from Open Philanthropy Project to Georgetown University.

### Competing interest statement

The authors declare no conflict of interest.

### Additional information

No additional information is available for this paper.
